# Evolution in *Sinocyclocheilus* cavefish is marked by rate shifts, reversals, and origin of novel traits

**DOI:** 10.1186/s12862-021-01776-y

**Published:** 2021-03-17

**Authors:** Ting-Ru Mao, Ye-Wei Liu, Madhava Meegaskumbura, Jian Yang, Gajaba Ellepola, Gayani Senevirathne, Cheng-Hai Fu, Joshua B. Gross, Marcio R. Pie

**Affiliations:** 1 Guangxi Key Laboratory for Forest Ecology and Conservation, College of Forestry, Guangxi University, Nanning, Guangxi People’s Republic of China; 2 Key Laboratory of Environment Change and Resource Use, Beibu Gulf, Nanning Normal University, Nanning, Guangxi People’s Republic of China; 3 Faculty of Science, University of Peradeniya, Peradeniya, Sri Lanka; 4 Department of Organismal Biology & Anatomy, University of Chicago, Chicago, IL USA; 5 Department of Biological Sciences, University of Cincinnati, Cincinnati, OH USA; 6 Departamento de Zoologia, Universidade Federal do Paraná, Curitiba, PR Brazil

**Keywords:** Phylomorphospace, Evolutionary convergence, Blind fish, Troglobitic, Geophylogeny

## Abstract

**Background:**

Natural model systems are indispensable for exploring adaptations in response to environmental pressures. *Sinocyclocheilus* of China, the most diverse cavefish clade in the world (75 species), provide unique opportunities to understand recurrent evolution of stereotypic traits (such as eye loss and sensory expansion) in the context of a deep and diverse phylogenetic group. However, they remain poorly understood in terms of their morphological evolution. Therefore, we explore key patterns of morphological evolution, habitat utilization and geographic distribution in these fishes.

**Results:**

We constructed phylogenies and categorized 49 species based on eye-related condition (Blind, Micro-eyed, and Normal-eyed), habitat types (Troglobitic—cave-restricted; Troglophilic—cave-associated; Surface—outside caves) and existence of horns. Geometric-morphometric analyses show Normal-eyed morphs with fusiform shapes segregating from Blind/Micro-eyed deeper bodied morphs along the first principal-component axis; second axis accounts for shape complexity related to horns. The body shapes showed a significant association with eye-related condition and horn, but not habitat types. Ancestral reconstructions suggest at least three independent origins of Blind morphs, each with different levels of modification in relation to their ancestral Normal-eyed morphs; *Sinocyclocheilus* are also pre-adapted for cave dwelling. Our geophylogeny shows an east-to-west diversification spanning Pliocene and Pleistocene, with early-diversifying Troglobitic species dominating subterranean habitats of karstic plains whereas predominantly Surface forms inhabit hills to the west. Evolutionary rates analyses suggest that lineages leading to Blind morphs were characterized by significant rate shifts, such as a slowdown in body size evolution and a 5–20 fold increase in rate of eye regression, possibly explained by limited resource availability. Body size and eye size have undergone reversals, but not horns, a trait entailing considerable time to form.

**Conclusions:**

*Sinocyclocheilus* occupied cave habitats in response to drying associated with aridification of China during late Miocene and the Pliocene. The prominent cave-adaptations (eye-regression, horn-evolution) occur in clades associated with the extensive subterranean cave system in Guangxi and Guizhou provinces. Integration of morphology, phylogeny, rate analyses, molecular-dating and distribution show not only several remarkable patterns of evolution, but also interesting exceptions to these patterns signifying the diversification of *Sinocyclocheilus* as an invaluable model system to explore evolutionary novelty.

**Supplementary Information:**

The online version contains supplementary material available at 10.1186/s12862-021-01776-y.

## Background

Due to the absence of light, absence of primary productivity, frigid temperatures and paucity of dissolved oxygen, subterranean habitats are among some of the most challenging environments for life on earth [1, 2]. From surface-dwelling ancestral species, cavefish have secondarily adapted to live in cave systems, often demonstrating a remarkable array of morphological and behavioral adaptations [3, 4]. These involve enhanced sensation, and also dispensing of traits that incur a developmental or energetic cost. Cavefish species can be divided into two forms—Troglophilic closely associated with caves, but not entirely dependent on them, and Troglobitic, the obligate cave dwellers [5, 6]. Troglobitic species may bear special adaptations, such as complete eye loss, loss of pigmentation, changes in cranial symmetry, proliferation of neuromast sensory organs, development of horns, and in some species flat, hollow heads [7–9]. Despite the ~ 200 cavefish species being known from across the world, large diversifications of cavefishes are rare. However, one extensive diversification occurs in *Sinocyclocheilus* (Cyprinidae, Barbinae), a monophyletic group of cyprinid fishes endemic to China, which allows a robust analysis of trait evolution relative to troglomorphism in a phylogenetic context.

The specialized traits cavefish bear have led them to be investigated as models of evolution, especially with respect to adaptations to novel environments and evolutionary convergence [5, 7, 10–15]. A lion's share of knowledge on evolution and development in cavefishes has come from *Astyanax mexicanus* (Mexican tetra), a species with both surface-dwelling (pigmented and eyed) and cave-dwelling morphs (depigmented and blind), which readily interbreed [16]. In contrast to this well-studied model, *Sinocyclocheilus* species not only include blind and normal-eyed morphs [17], but demonstrate a continuum from blind to normal-eyed species. Indeed, members of the *Sinocyclocheilus* genus display remarkable morphological evolution with divergent cave-dwelling, cave-associated, and surface-dwelling species.

*Sinocyclocheilus* species are thought to have shared a common ancestor in the late Miocene, undergoing a spectacular diversification spanning the Pliocene and Pleistocene, across the southwestern parts of China’s 620,000 km^2^ of karst habitats [18], with nearly 75 extant species [19]. This resulted in an adaptive diversification into subterranean refugia traversing the intersection of the Guizhou, Guangxi and Yunnan provinces around the time of the uplifting of Tibetan/Guizhou plateau [12].

One of the most striking forms of cave adaptation in *Sinocyclocheilus* is variation in eye morphology, categorized often into three morphs [20], ranging from Normal-eyed, through Micro-eyed (small-eyed) to Blind species. Of all Chinese hypogean fishes, 56 species show troglomorphism such as reduction and/or loss of eyes, pigmentation, and the gas bladder. Presence of a horn-like structure and hyper-development of the dorsal protuberance (humped back) are two additional unique characters to certain Chinese hypogean species [21]. These dramatic adaptations to cave life are reflected in the unique morphology of these fish.

While the morphology of *Sinocyclocheilus* is likely attributed to their habitat and local adaptations, the precise function of certain morphologies (e.g., their horns) remains unknown [22]. For instance, many blind species are obligate cave dwellers that have the ability to navigate along cave walls, cave-bottoms and within narrow passages [4]. Yet, others are open-water species that navigate in the manner typical of fish. There are also intermediate forms between these two principal morphs [20]. However, the morphology of these fishes is so extreme and substantial variation in morphology is evident even within blind, intermediate and open-water species. We chose to examine evolution of shape related variation of these fishes using mainly geometric morphometrics and ancestral state reconstructions.

Much of the work on *Sinocyclocheilus* has been taxonomic in nature [23], and some species are only known from a few specimens or photographs [23–31], and in some cases, only from a single type specimen [19]. Over the past decade, however, the inventory of *Sinocyclocheilus* species has steadily increased [6, 19, 20, 23–25, 28–39], indicating that there are more species yet to be discovered. Difficulties in sampling deep caves scattered across the karstic expanse, and the extreme rarity of some species have impeded exploratory and monitoring studies. Hence we build on previous data (genetic, morphology and distribution), together with the data that we accumulated over 3 years.

*Sinocyclocheilus* is an emerging model system and studies have explored gene mapping, eye degeneration, and mechanics of the lateral line system [14, 40–42], albeit in only a few handpicked species from across the phylogeny. However, future studies would benefit from a comprehensive analysis of the evolution of major traits of the *Sinocyclocheilus* diversification and would bolster both comparative work and hypothesis testing in this group of fishes.

Here, we explore key patterns of morphological evolution, habitat utilization and geographic distribution in these fishes. We ask the following main questions: What are the patterns of evolution of morphological diversity and habitat utilization? What is the geographic distribution and chronology of this diversity?

We show evolution in *Sinocyclocheilus* to have been associated with convergence, significant rate changes and trait reversals across their phylogenetic history and that adaptations for cave dwelling originated in the early-emerging clades inhabiting the eastern expanse of their distribution.

## Results

### Phylogenetic inference and ancestral state reconstruction

**Fig. 1:**
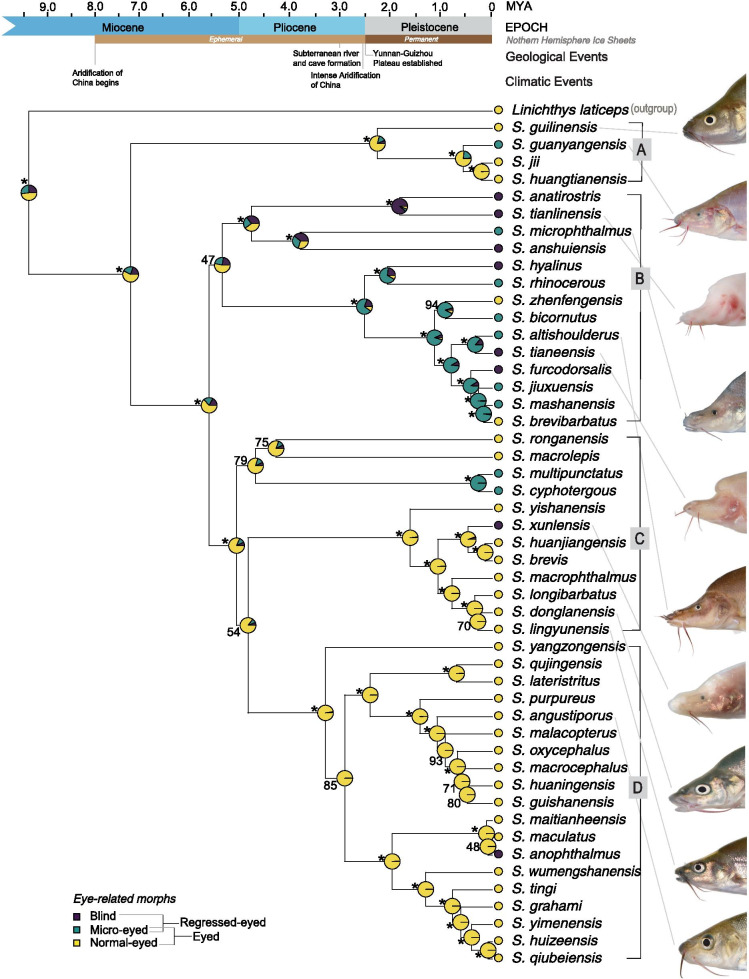
Ancestral character state reconstruction using stochastic character mapping for the eye-trait morphology (Blind, Micro and Normal-eyed morphs) on a time calibrated phylogeny of using a “standard clock” estimate. A, B, C and D are four major clades. Posterior probabilities of node support values of 100% are indicated by *. Key events of *Sinocyclocheilus* evolution includes; at least three independent evolutionary events for Blind morphs; Blind, Micro-eyed and a few Normal-eyed species in Clade B, with two cases of reversal from either Micro-eyed or Blind to Normal-eyed species. *Sinocyclocheilus* diversification seems to have initiated in the mid-Miocene with the aridification of China

The maximum credibility tree of *Sinocyclocheilus* is shown in Fig. 1, together with the reconstruction of ancestral states for eye-related morphs; we consider four major clades (A, B, C, D), as previously reported by other authors [20]. Despite the uncertainty inherent in ancestral state reconstructions, it is clear that Blind species evolved at least three times in *Sinocyclocheilus*. Two of these events involved single species evolving from Normal-eyed ancestors, namely *S. xunlensis* and *S. anophthalmus*. On the other hand, the third lineage of blind *Sinocyclocheilus*, Clade B, includes several closely related species of Blind, Micro-eyed and a few Normal-eyed species, with two cases of reversal from either Micro-eyed or Blind to Normal-eyed morphs, namely *S. zhenfengensis* and *S. brevibarbatus* (Fig. 1). All four clades contain Regressed-eyed species and comparatively, Clade B contains most cases with Regressed-eyed species. Our analyses indicate that Clade B originated in the early Miocene about 5.5 million years ago, whereas the other two transitions to blind species were much more recent. Our research result also shows that most recent common ancestor of *Sinocyclocheilus* originated about 7.4 million years ago (Fig. 1). However, in the approach using two calibration points to estimate divergence time, Clade B seems to have originated about 6 million years ago, and the divergence time between the species in Clade A and other species in the genus *Sinocyclocheilus* is about 8.5 million years ago (Additional file 1: Fig. S1).

Ancestral state reconstruction of habitat type indicated (C) that troglobitic ancestral species have evolved first and surface dwellers evolved later, especially the species in Clade D. Few species in clades B and C (*S. brevibarbatus*, *S. multipunctatus*, *S. macrolepis* and *S. longibarbatus*) show reversals from troglobitic to troglophilic recently in their evolution whereas species in Clade D evolved to predominantly a surface habitats since the Pliocene. Further, *S. anophthalmus* in Clade D exhibit a second event of cave origin fairly recently in the phylogeny.

In our study, horned morphs have originated from a non-horned ancestor and are restricted to Clade B where they exhibit a pattern such that sister taxa of a horned morph is a non-horned species in most cases (Additional file 1: Fig. S2).

### Patterns of morphological trait evolution and phylomorphospace analysis

**Fig. 2:**
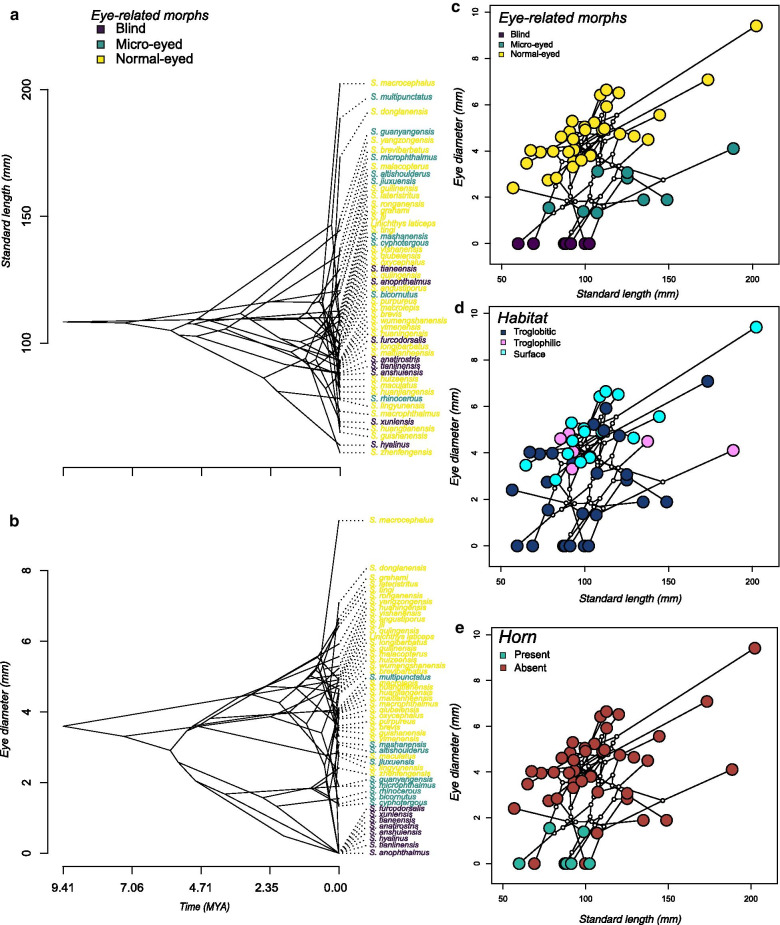
Temporal patterns of body size and eye diameter evolution and the other three traits (Horn, Habitat, Eye-related morphs) against standard lengths as phylomorphospace-traitgrams in *Sinocyclocheilus*. The traitgrams suggests that the evolution of different morphs is attained by altering the allometric relationships between body size and eye diameter. **a** Traitgram of body size; **b** Traitgram of eye size; **c** Eye related morphs traced on the phylomorphospace indicating clear separation of the three morphs in the morphospace. **d** Habitat associations traced on the phylomorphospace showing species having eye diameter < 3 mm and small to medium body sizes are obligate cave dwellers whereas species with eye diameter ≥ 3 mm can be Troglobitic, Troglophilic or Surface dwellers regardless of their body size. **e** Horn related morphs traced on the phylomorphospace indicating the presence of a horn in smaller blind fish and smaller fish with reduced eye size. Horned species are all Troglobites

Our analyses shows that the evolution of body size and eye diameter in *Sinocyclocheilus* showed rather distinct dynamics (Fig. 2). For instance, body size evolved at a relatively constant rate early in the evolution of the genus, with an apparently accelerated rate near the present, but with little correspondence with eye morphs. Indeed, normal-eyed species span the entire range of body sizes (Fig. 2a). On the other hand, there was an early differentiation in eye diameter at around 4.7 Mya between normal and micro-eyed/blind species, but many other reversals took place near the present (Fig. 2b, c). These patterns suggest that eye and body size evolution in *Sinocyclocheilus* were largely decoupled during its history. Habitat associations traced on phylomorphospace (Fig. 2d) indicate that species with regressed eyes and small-to-medium body sizes are obligate cave dwellers. This same pattern is shown by the standardized eye diameter related trigrams also (Additional file 1: Fig. S3). However, normal eyed species can be Troglobitic, Troglophilic or surface dwellers regardless of their body size. Our results indicate that all horned species are obligate cave dwellers while not all cave species are horned (Fig. 2e).

**Fig. 3:**
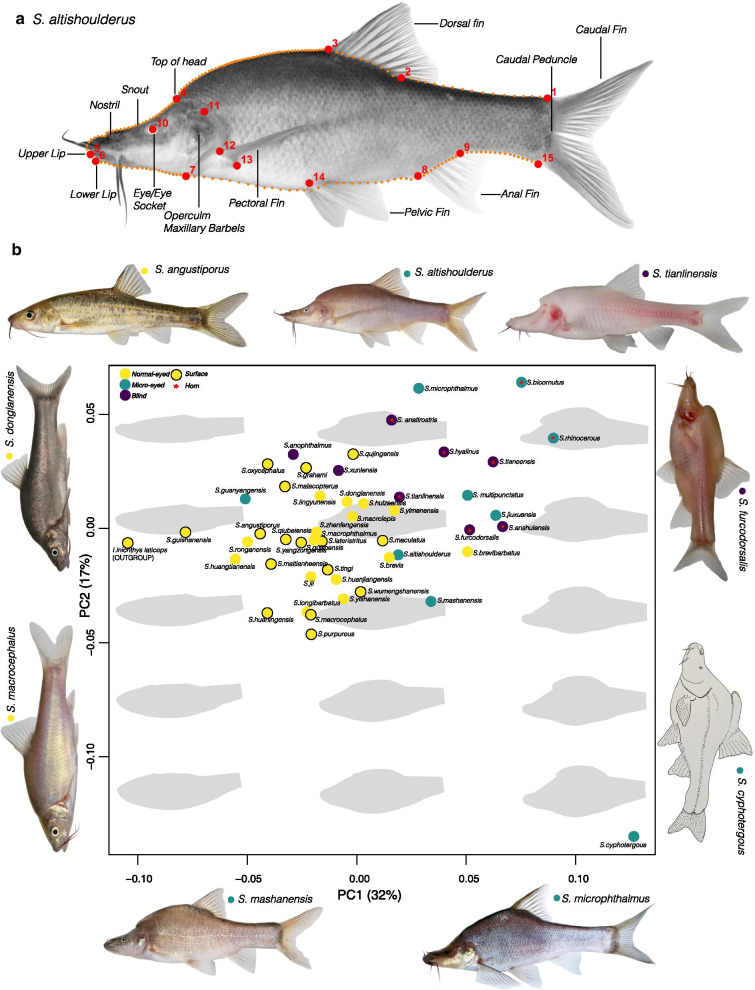
**a** A specimen of *Sinocyclocheilus altishoulderus* indicating the position of 15 landmarks (red: larger points indicated by numbers 1–15) and 180 semi-landmarks (Orange: smaller point) used for the calculation of Procrustes coordinates and traditional linear measurements (*SL* standard length, *ED* eye diameter and *sED* standardized eye diameter) used in the geometric morphometric analyses. **b** PCA showing the variation in body shape of the genus *Sinocyclocheilus* traced with eye morphology, habitat occupation, existence of horn. PC1 and PC2 accounts for 32% and 17% of the variance respectively. A shift from the fusiform shape of the Normal-eyed surface forms to a more “boxy” form of the Micro-eyed and Blind forms is evident

A more precise description of the overall changes associated with different morphs can be visualized in the projections built from the geometric morphometrics analyses (Fig. 3b). The first PC, which accounted for approximately 32% of the variance in the dataset (see Additional file 1: Table S1), tended to distinguish the slender Normal-eyed species on the left and Micro/Blind species on the right, which were characterized by changes in shape and widening of the anterior dorsal area between mouth and beginning of the dorsal fin of their body, resulting in a shift from the fusiform shape of the Normal-eyed forms to a more “boxy” form of the Micro-eyed and Blind forms. The second PC, which explained approximately 17% of the variance in the dataset, emphasized the differences in the type of dorsoventral broadening of the mid-section between morphs, with a shortening of the tail region (Fig. 3b). The variation in this axis is very high among the Micro-eyed and Blind forms when compared to the Normal morphs. Multivariate Analysis of Variance (MANOVA) showed that Eye-morphology and Horn bearers were significantly different in body shape; Body shape was not significantly associated with habitat type. (Eye-morphology, F = 15.28, p = 0.001; Horn, F = 6.14, p = 0.016; Habitat, F = 1.72, p = 0.153).

### Rates of morphological evolution

The multiple-rate model of evolution provided the best fit to the data for all three quantitative traits (ΔAIC = 3.6, 14.2 and 8.3 respectively for ED, sED and SL; Table 1), indicating that the evolution of different *Sinocyclocheilus* morphs was associated with significant changes in their evolutionary rates. However, there were intriguing differences between traits in their rates (Table 2). The rates of evolution of eye diameter and standardized eye diameter were similar between Normal-eyed and Micro-eyed species, but increased between 5.5 and 20.8 times during shifts towards Blind species.

**Table 1:** Model fit and estimated Brownian rate parameters for three traits (ED, sED and SL) in eye related morphs of *Sinocyclocheilus*

Trait	Single rate model		Multiple rate model		ΔAIC
	AICc	AIC weights	AICc	AIC weights	
ED	254.9848	0.142077010	251.3885	0.857923	3.596290
sED	249.0874	0.000806064	234.8423	0.999194	14.245083
SL	532.7615	0.015145208	524.4119	0.984855	8.349620

Multiple-rate models of evolution providing the best fit to the data for all three quantitative traits with ΔAIC > 2

**Table 2:** Model averaged rate parameters for the measured traits in eye related morphs of *Sinocyclocheilus*

Trait	Model averaged rate		
	Blind	Micro-eyed	Normal-eyed
SL	44.0468	1775.2360	1465.4660
ED	23.2990	3.9025	4.5091
sED	44.6336	1.4413	2.8972

Normal-eyed and Micro-eyed species indicate similar evolutionary rates with marked shifts towards Blind species

### Spatial patterns in *Sinocyclocheilus* evolution

**Fig. 4:**
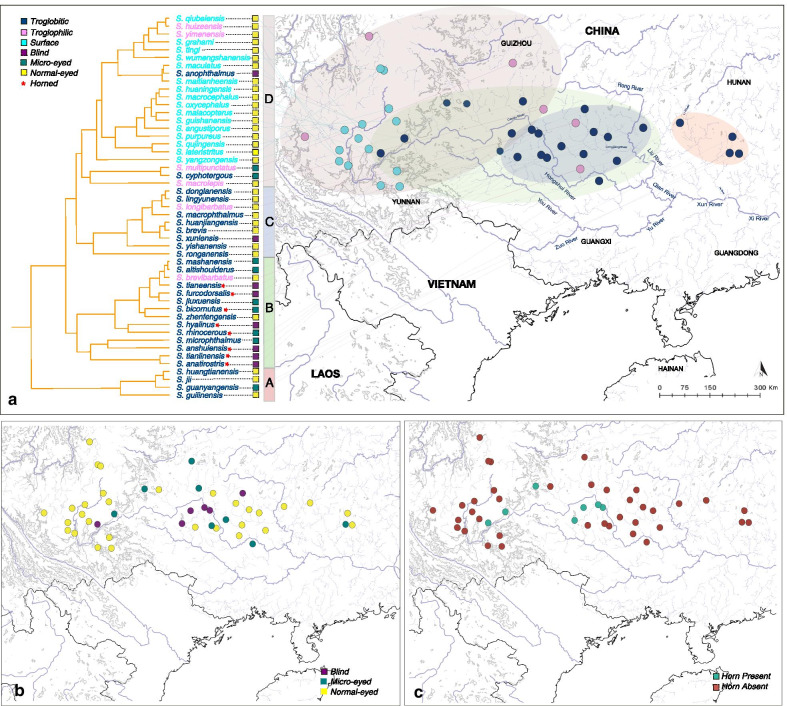
Geophylogeny, the phylogeny laid across the geographic distribution of the species considered in the analysis, with habitats (**a**), eye-related morphs (**b**) and horn-existence (**c**). A pattern where early-diverging, Normal-eyed, Troglobitic species are placed in the east, predominantly Blind/Micro-eyed/Normal-eyed, Troglobitic species in the center and Normal-eyed, Surface dwelling species towards the western mountains is evident, indicating an east to west dispersion of the genus *Sinocyclocheilus* across South and South western China. Eye specializations mostly occurred in Clade B, and horn evolution occurred exclusively in Clade B, within the Central range of the *Sinocyclocheilus* distribution. [Vector files were obtained from a public domain map data at http://naturalearthdata.com]

Geophylogeny represents the phylogeny overlaid across the geographic location of each species, where phylogenetic clustering is evident across the landscape. Considering the distribution of *Sinocyclocheilus*, we see mainly a pattern where the early-diverging, Normal-eyed morphs are placed in the east, a substantial portion of Blind/Micro-eyed (Regressed-eyed) species are in the center, and Normal-eyed morphs are predominant towards the western mountains (Fig. 4a and b). Although few in numbers, horned species (all horned species are contained in Clade B) seems to be scattered within their distribution range (Fig. 4c).

## Discussion

### Habitat utilization in context of eye-morphology

Integrating evolution of eye size and habitat manifests interesting and previously unrecognized evolutionary patterns in the evolution of *Sinocyclocheilus*. The eyed-size based ancestral reconstruction suggests the base of the phylogeny is most likely an eyed species (i.e. Normal- or Micro-), but habitat reconstructions places, with high probability, Troglobitic species at the base (Additional file 1: Fig. S4). This suggests an ancestral eyed-species evolved a Troglobitic habit before becoming blind. This may be an example of preadaptation in *Sinocyclocheilus*, i.e., the advancement of a functional change with little or no evolutionary modification [43]. In *Astyanax* cavefish, surface-dwelling forms are scotophilic; they prefer to remain away from direct light, suggesting that scotophilia may be preadaptive for colonizing the dark cave environment [44], but there are no confirmed cases of character reversal in *Astyanax*. In *Sinocyclocheilus*, since an ancestral (eyed) species demonstrated preference for the cave habitat, this preadaptation to darkness may hint towards why certain species tend to become cave-dwellers while others do not. This pattern is supported by two principal lines of evidence. First, most of the early-diverging species are eyed, and Troglobitic (except for one species with an unusual eye-related polymorphic condition that we discuss below). Second, the most westward group (Clade D; Normal-eyed Surface fish), re-colonized caves whenever cave habitats were available within that area, suggesting a strong predisposition for cave-dwelling across all *Sinocyclocheilus*. In other words, when caves were available, members of *Sinocylocheilus*, irrespective of eye-related condition, prefer cave dwelling.

In the context of *Sinocyclocheilus*, the preference for caves may be not only a preference for darkness but also a preference for depth, in search of retreating water for survival. In karstic environments, where surface desiccation is common, a preference for such deeper refuges may have conferred a fitness dividend. In the presence of an array of subterranean waterways, such a predisposition would have given rise to the eye-regressed forms living close to, or associated with, caves that are characteristic of the genus.

Furthermore, apart from the Troglobitic and Troglophilic species of Clade D, some of the putative Surface species of this clade are often observed at the entrances of caves or at windows to subterranean rivers [20]. Future ecological studies may reveal that some taxa recognized as Surface forms may indeed be Troglophilic, bolstering the notion that *Sinocyclocheilus* are predisposed to seek refuges in deeper waters of karstic caves.

Resource utilization plays a key survival role in harsh environments [45], as maybe the case for *Sinocyclocheilus*. Some Troglophilic eyed-species like *S. altishoulderus, S. donglanensis* [21], *S. microphthalumus*, *S. malacopterus* [46] and *S. longibarbatus* (personal observation), are known to emerge from caves during the high-water season, presumably to feed and breed. This explains dependence on the cave as a diurnal refugium, from where these species can exploit the surface habitats at night. Strategies such as this, where multiple resources are utilized concurrently, points to the adaptability of some *Sinocyclocheilus* species, resulting in their persistence in a harsh and changing environment. In this sense, the cave entrances are possibly an ecotone that is important during *Sinocyclochelius* diversification.

### Adaptations in the light of geophylogeny

Early-diverging *Sinocyclocheilus* (Clade A) are Normal-Eyed, predominantly cave dwelling and non-horned species from the eastern region of their distribution. The ancestral morphs (at the base) suggest that the affinity to caves would have evolved early and is present in most *Sinocyclocheilus.* The clade comprising of early-diverging species is from the east of the *Sinocyclocheilus* distribution, i.e. the He Jiang and Gui Jiang basin in northeastern Guangxi, and hence, it seems that the diversification of these fishes occurred from east to west (Fig. 4a).

Within this predominantly Normal-eyed clade (Clade A), there is an exception: *Sinocyclocheilus guanyangensis*, a species that we coded as Micro-eyed, has Normal-Eyed, Micro-eyed and effectively Blind morphs within the same population, thus it is polymorphic for this trait. But these blind morphs have their eyes completely covered by skin and the eye is not itself degenerate. This kind of condition has been observed also in several other congeners (*S. xunlensis* and *S*. *flexuosdorsalis—*latter species not included in our analysis due to lack of molecular data), but is uncommon. This suggests a degree of polymorphism for this trait, indicating that the earliest ancestors of *Sinocyclocheilus* may have been able to lose or gain eyes relatively easily as an adaptation to local conditions. This pattern appears several times within this cave-driven diversification.

The major adaptation for cave dwelling evolves predominantly in the expansive karstic area in northwestern Guangxi (associated with the Liu Jiang basin and Hongshui river basin joining the main Xijiang River system from the North), in Clade B, the southeastern corner of Guizhou province (upper reaches of Hongshui River) and the northeastern plateau of Yunnan province. This region can be considered the center for novel adaptations for *Sinocyclocheilus*, where these fishes express their full morphological diversity, blindness, micro-eyedness, and their remarkable horns. In the shape-related analyses, these species cluster on the right of morphospace (Fig. 3b). The deeper caves and extensive subterranean river system associated with the Guangxi plains [20] would have facilitated this extensive adaptive diversification (Fig. 4).

The karstic northwestern region to which the Guangxi-dominated clade (Clade B) belong to experiences drought conditions during much of the year, and one of the major sources of rain for the region is from storms sweeping from the southeast, which are strong enough to persist through the vast plains of Guangxi, mainly from April to August [20]. During the intervening dry periods, however, these fishes seem to have found refuge in the subterranean caves. The morphologically most diverse clade inhabiting the region where the climatic conditions are most unfavorable to surface fish reinforces the notion that *Sinocyclocheilus* adapted to life in caves as climatic refuges [20].

The distribution of Clade C, characterized by mostly Normal-eyed but Troglobitic species largely overlaps Clade B. In Clade C, the single Blind species (*S. xunlensis*) and the two Micro-eyed species (*S. cyphotergous* and *S. multipunctatus*) are shown within a narrow geographic area on the Liu Jiang and Hongshui river system (Fig. 4b).

The clade containing species to the west (Clade D), predominantly on the hilly terrain of Yunnan plateau, are Normal-eyed Surface species lacking horns (Figs. 1 and 4). However, when cave habitats and subterranean river systems are available, some of these surface adapted species have become Troglobytic or Troglophilic. The Troglobytic species occurring in the region, *S. anophthalmus*, is blind. However, this Blind species stands clustered with the Normal-eyed morphs in morphospace, signifying that the shape of the species has not extensively changed, possibly due to recent (Pleistocene) invasion of the cave habitat by a Normal-eyed ancestor (Fig. 1) suggesting that the time since entering the cave has been insufficient to change into the characteristic box-like shape of the Blind species of Clade B.

Horn distributions show several peculiar trends. In most *Sinocyclocheilus* species, a prominent hump is present [47]. However, this hump is markedly low in the Normal-eyed surface-inhabiting species of the Yunnan clade (Fig. 4). For species that bear a horn, this structure occurs in the region in which the dorso-frontal hump is present, and always occurs anterior to the hump, at the dorsal occipital margin. The exception to this is *S. cyphotergous* (Clade C), where a horn-like structure occurs close to the anterior end of the dorsal fin, on top of the hump. This species (in Clade C) is phylogenetically separate from other horned species, suggesting that the origins of the “horn” for this species is evolutionarily different from the other horned species (Additional file 1: Fig. S2). Though the function of the horn remains unknown, functions such as protection of head, a role in sexual selection, and anchoring in strong current have been suggested [20, 48]. In *S. cyphotergous*, this structure may be similar to those in other species in function.

### Troglomorphism and rates of evolution

The rates of evolution of various traits show some incongruent (non-allometric, *R* = 0.034, *p* = 0.82), but interesting patterns that can be explained in the context to adaptations to a Troglobitic condition. The rates of evolution in eye diameter are similar between Normal- and Micro-eyed species but, increase dramatically (5.5–20.8 times) with shifts towards blind forms. However, body size evolution for these morpho-groups shows a reversed pattern, with a 0.03 decrease in body size evolution in the Blind morphs compared to the eyed-morphs. These patterns in rate variation suggest that the evolution of Blind morphs to a Troglobitic habitat were simultaneously associated with an increase in the rate of evolution of eye-size degeneration and a decrease in the rate of body size evolution. The smaller body size resulting from a sluggish rate of change facilitates both navigation within constricted spaces and sustenance on a limited supply of resources as expected in subterranean habitats [49].

Much of our collective knowledge of the patterns and mechanisms of regressive evolution come from studies of animals that have colonized the subterranean biome. Within this group, several studies have focused on the *Astyanax mexicanus* [5, 50–53]. This natural animal model system comprises multiple cave-adapted morphs and a surface-dwelling morph that resides in or near the caves themselves [54]. Since the discovery of this cavefish in 1936, studies have provided insight to the developmental and genetic bases for cave-associated traits [55–62]. Indeed, much of this insight has emerged from the interbreeding studies of conspecific cave and surface morphs (reviewed in [63]). However, several aspects of regressive evolution and troglomorphic adaptation remain unresolved. Owing to several of the differences with *Astyanax*, we argue that *Sinocyclocheilus*, in the form of a multi-species evolutionary model system, is well-positioned to provide important new insights to broader patterns of diversification and adaptation in cave-dwelling organisms.

In conclusion, *Sinocyclocheilus* fishes seem to have occupied the cave habitats in response to drying associated with aridification of China during late Miocene and the Pliocene. The early-diverging clades of these fishes are located in the eastern corner of their distribution, predominantly in Guangxi region. The most prominent cave-adaptations such as reduction and loss of eyes and evolution of horns also seem to have taken place in clades that are predominantly found in Guangxi and Guizhou provinces and are associated with the extensive subterranean cave system in the area. There is eye-related and Horn related two-dimensional shape variation seen within the diversification, which is statistically significant. The habitat related shape variation is not significant, this is because Normal-eyed species are in most clades are also troglobitic, except in Clade D, the westward clade in this diversification. The lineages leading to Blind morphs are associated with significant rate shifts such as slow down of body size evolution and significant increase of eye-regression; this is possibly associated with the scarcity of resources. The integration of morphology, phylogeny, rate analyses, dating and distribution show not only several remarkable patterns of evolution, but also interesting exceptions to these patterns, which signify the diversification of *Sinocyclocheilus* as a unique multi-species model system to study evolutionary novelty.

## Methods

We carry out an analysis to explore the key patterns of morphological evolution, habitat utilization and to determine how these patterns are dispersed across the landscape in *Sinocyclocheilus* cavefishes. We first infer a phylogeny based on mitochondrial DNA (mtDNA) which we use to determine patterns of evolution. We also determine shape associations of these species in terms of eye morphology, habitat and horn existence using a landmark based analysis. We calculate the rates of evolution of body size in relation to eye size evolution, habitat utilization and horn evolution. We use ancestral reconstructions on a timing-tree to determine the evolutionary patterns of eye regression, habitat occupation and horn evolution. Finally we trace the how these patterns are dispersed across the landscape using a geophylogeny. This framework will provide a comprehensive understanding of the major patterns of morphological evolution for *Sinocyclocheilus* cavefishes.

### Phylogeny estimation

We compiled sequence data from GenBank for two mtDNA fragments (NADH4 and cytb) of 39 *Sinocyclocheilus* species, and five outgroup species *Linichthys laticeps*, *Gymnocypris eckloni*, *Gymnocypris przewalskii*, *Labeo batesii*, *Puntius ticto*. In addition, we generated sequence data for the NADH4 and cytb gene fragments of nine additional *Sinocyclocheilus* species (Additional file 1: Table S2). For these species, total genomic DNA was extracted using the DNeasy Blood and Tissue Kit (Qiagen Inc., Valencia, CA) following the manufacturer’s protocols. DNA was amplified in 25-µL volume reactions: 3 mM MgCl_2_, 0.4 mM of dNTP, 1X buffer, 0.06 U of Taq DNA Polymerase, 2 mM of each primer. Thermocycling conditions included an initial step at 94 °C for 3 min, followed by 35 cycles at 45 s at 94 °C, 1 min at 46–50 °C and 45 s at 48–56 °C, and a final step at 72 °C for 5 min. PCR products were electrophoresed in a 1.5% agarose gel, stained with ethidium bromide and visualized under UV light. Successfully amplified products were purified using MicroconTM Centrifugal Filter Units (Millipore, Billerica, MA, U.S.A.). Sequencing reactions were carried out in 10 µl solutions including the following final concentrations: 5 ng/µl of template DNA, 0.5 µl of Big DyeTM (Applied Biosystems Inc., Foster City, CA, U.S.A.), 0.2 µM of each primer and 0.1X of reaction buffer. The final product was purified using SephadexTM G-50 (GE Healthcare Bio-Sciences AB, Uppsala, Sweden) for sequencing. Forward and reverse strands were reconciled using Staden v.1.6.0 [64].

Sequences from both genes were concatenated and aligned unambiguously using ClustalW [65], as implemented in MEGA v. 6.0 [66], for a total alignment length of 2167 bp. We used jModelTest v.2.1.10 [67] to determine the best models of evolution for each fragment, which were implemented in BEAST v.1.10.4 [68] as a partitioned analysis to estimate the phylogenetic relationships and relative divergence times within *Sinocyclocheilus*. Due to the lack of a fossil record for the *Sinocyclocheilus*, we used two alternative approaches to estimate divergence times in *Sinocyclocheilus*. First, we used a “standard clock” estimate for fish mitochondrial DNA of 2% per million years [19], as the main approach of this study. We also used two calibration points (C1, C2) based on the approach of Li et al. [12] for exploring potential differences in estimates of divergence times using different approaches. We used a strict molecular clock and a calibrated Yule tree prior, as well as a GTR + I + G for each partition and ran the analysis for 20 million generations using the Cipres Science Gateway Server [69]. Convergence was assessed by inspecting the log-output file in TRACER v.1.7.1 [70] and by ensuring ESS values were above 200. The first 10% of the trees were discarded, and the post burn-in trees were used to infer the maximum clade credibility tree using TreeAnnotator v.1.10.4 [71]. The maximum clade credibility tree, as well as a set of 1000 post burn-in topologies, were retained for further analyses (see below).

### Morphometric data acquisition and analyses

To determine shape associations of these species, we assembled a database of images of referenced specimens of adult *Sinocyclocheilus* from scaled photographs (we only used images that contained scale-bars), which were complemented by species that we photographed (Additional file 1: Table S3), and we measured the left side (lateral view) of the fish. The final dataset included 90 images (54 photographs by us and 36 from photographs from previous studies) for 50 species, which included 65%of the total number of described *Sinocyclocheilus* species. These images were used for geometric morphometrics analyses, which were based on 15 landmarks (Fig. 3a) and 180 sliding semi-landmarks, obtained using tpsDig v. 2.16 [72]. Semi-landmarks were collected as curves outlining the body. These data were subsequently reduced to equidistant landmarks, and defined as semi-landmarks using tpsUtil v. 1.46. These images were treated with the Unbend function of tpsUtil to remove bending effects [73]. We then slid the landmarks using the bending energy method [74] implemented in GEOMORPH v.3.2.0 [75]. The landmark coordinates were aligned using a generalized Procrustes superimposition analysis [76], and a principal component analysis (PCA) was used to evaluate shape variation within the sample. Multivariate analysis of variance (MANOVA) was performed to determine if there were statistically significant differences in body shape among the below mentioned categorizations tested (Eye-morphology, Habitat and Horn).

Multiple images for the same species were used to obtain the landmarks (1–3 photographs per each species, Additional file 1: Table S3) and the mean of their Procrustes coordinates were calculated to be used in later analyses. We also obtained traditional linear measurements, namely standard length (SL), eye diameter (ED) and standardized eye diameter (sED, calculated as the ratio between ED and SL). We used zero for the eye diameter for the blind fishes.

### Morphological and habitat evolution

Since shape variation in *Sinocyclocheilus* cavefishes occurs mostly in the anterior end of the fish and as one of the major features leading to this is eye-related, we considered the absence or the size of the eye (when present) as a proxy for the categorization of morphs. Since the eye size has an allometric association with body size, we used the standardized eye diameter (sED) in placing them into three morphological categories: Blind (eye absent); Micro-eyed (≤ 3.0 mm); Normal-eyed (> 3.0 mm) respectively. For ease of discussion, we considered Blind and Micro-eyed together as Regressed-eyed; Micro-eyed and Normal-eyed together as eyed species.

*Sinocyclocheilus* were also categorized based on their habitat as Troglobitic, Troglophilic, and Surface species. Troglobitic species live in an obligatory association with caves and are not sampled outside of caves. Caves, as meant here represent roofed-caves, submerged caves, and subterranean waterways that form windows intermittently with the surface. Troglophilic species live in a close association with caves and are sampled both in the vicinity of cave entrances and within caves. Finally, Surface species are found in habitats even when a cave is not found in close proximity and live in normal streams ponds and lakes as typical fish do, but they could venture into caves (underground water bodies) during unfavorable periods, when water is only available in caves. It should be noted here that this categorization is strictly habitat based and not morphology based (for instance, there are Normal-eyed species that are Troglobitic, Troglophilic or Surface). These habitat associations are based on published literature [20, 21] and personal observations as outlined in Additional file 1: Table S2.

To infer the number and timing of evolutionary shifts within eye-related morphs, horn distribution and the habitat type, we used stochastic character mapping [77, 78], as implemented in the make.simmap (model = "ER") function in PHYTOOLS. We tested multiple models for the ancestral state reconstruction, the ER model gave the highest likelihood. On each of the 1000 post burn-in trees obtained from BEAST, we used stochastic character mapping to generate 100 potential histories. This approach therefore considers uncertainty both in the evolutionary history of the traits as well as in the inferred topology of the phylogeny.

The landmark coordinates obtained were aligned using a generalized Procrustes superimposition analysis [76], and a principal component analysis (PCA) was used to explore shape variation within the sample. In addition, we described the eye-related morphological variation in *Sinocyclocheilus* by estimating ancestral states of SL and ED and visualizing them using traitgrams [79] as implemented in the phenogram function in PHYTOOLS [80] using the maximum clade credibility tree. We also visualized the evolution of both traits simultaneously using a phylomorphospace [81] using the phylomorphospace function in PHYTOOLS. Furthermore, to assess the allometric relationship of the size of the eye against the standard body length we also performed an allometric regression between SL and sED.

### Evolutionary rate variation in eye related morphs

We tested whether the evolutionary rates of the studied continuous traits (SL, ED, sED) are significantly different in different morphs. We used 100 potential trait histories from stochastic character mapping and then fit two alternative models of evolution on each studied trait, one that fixes the rate of evolution to be identical between morphs against an alternative model in which the morphs have separate rates. We calculated the Akaike Information Criterion for small sample size (AICc) from the maximum likelihood estimate on each tree using the brownie.lite function in PHYTOOLS. Finally, to incorporate uncertainty about model choice into the parameter estimate, we calculated model-averaged estimates of evolutionary rates for each morph using the Akaike weights from the mean AICc scores. Unless otherwise indicated, all analyses were conducted using R v. 3.6.2 [82].

### Geophylogeny analyses

To determine the patterns of evolution of eye-regression, habitat utilization and horn existence in the context of *Sinocyclocheilus* distribution we built a geophylogeny on which we traced these traits. We, however, could not carry out a formal biogeographical analysis, given that their high endemism and the complex pattern of underground connections between caves limits the establishment of reasonable biogeographical areas. However, we assessed the geographical structuring of *Sinocyclocheilus* diversification by building the geophylogeny on GenGIS v. 2.5.3 [83] based on the maximum credibility tree.

## Supplementary Information

**Additional file 1: Figure S1.** Ancestral character state reconstruction using stochastic character mapping for the eye-trait morphology (Blind, Micro and Normal-eyed morphs) on a time calibrated phylogeny. **Figure S2.** Ancestral character state reconstruction using stochastic character mapping for the horn related trait (presence/absence of horn) on the phylogeny of the genus *Sinocyclocheilus.* **Figure S3.** Temporal patterns of eye diameter evolution and the other three standardized traits (Horn, Habitat, Eye-related morphs) against standard lengths as phylomorphospace-traitgrams in *Sinocyclocheilus.* **Figure S4.** Ancestral character state reconstruction using stochastic character mapping for habitat occupation (Troglobitic, Troglophilic and Surface) on the phylogeny of the genus *Sinocyclocheilus*. **Table S1**. Calculated Principal Component values (PC1, PC2 and PC3) of all the specimens used in the current analysis. **Table S2.** Species information and GenBank accession numbers of two mtDNA fragments (*NADH4* and *cytb*) of 49 *Sinocyclocheilus* species. **Table S3.** Information of digitized images used in the morphometric geometric analysis

## Data Availability

All data generated or analyzed during this study or the sources of data (Genbank) are included in this published article.

## Abbreviations

mtDNA: Mitochondrial DNA
SL: Standard length
ED: Eye diameter
sED: Standardized eye diameter
PCA: Principal component analysis
MANOVA: Multivariate analysis of variance
AICc: Akaike information criterion
GXU: Guangxi University
