# Supplementary material for: Evolution in Sinocyclocheilus cavefish is marked by rate shifts, reversals, and origin of novel traits
Source: BMC Ecol Evol. 2021 Mar 17;21:45. doi: 10.1186/s12862-021-01776-y (PMC7968296; doi:10.1186/s12862-021-01776-y)
Supplement: Supplementary file 1 — Additional file 1: Figure S1. Ancestral character state reconstruction using stochastic character mapping for the eye-trait morphology (Blind, Micro and Normal-eyed morphs) on a time calibrated phylogeny. Figure S2. Ancestral character state reconstruction using stochastic character mapping for the horn related trait (presence/absence of horn) on the phylogeny of the genus Sinocyclocheilus. Figure S3. Temporal patterns of eye diameter evolution and the other three standardized traits (Horn, Habitat, Eye-related morphs) against standard lengths as phylomorphospace-traitgrams in Sinocyclocheilus. Figure S4. Ancestral character state reconstruction using stochastic character mapping for habitat occupation (Troglobitic, Troglophilic and Surface) on the phylogeny of the genus Sinocyclocheilus. Table S1. Calculated Principal Component values (PC1, PC2 and PC3) of all the specimens used in the current analysis. Table S2. Species information and GenBank accession numbers of two mtDNA fragments (NADH4 and cytb) of 49 Sinocyclocheilus species. Table S3. Information of digitized images used in the morphometric geometric analysis [file 12862_2021_1776_MOESM1_ESM.docx]

Additional Information for

**Evolution in *Sinocyclocheilus* cavefish is marked by rate shifts, reversals, and origin of novel traits**

**This includes:**

Figures S1 to S4

Tables S1 to S3

**
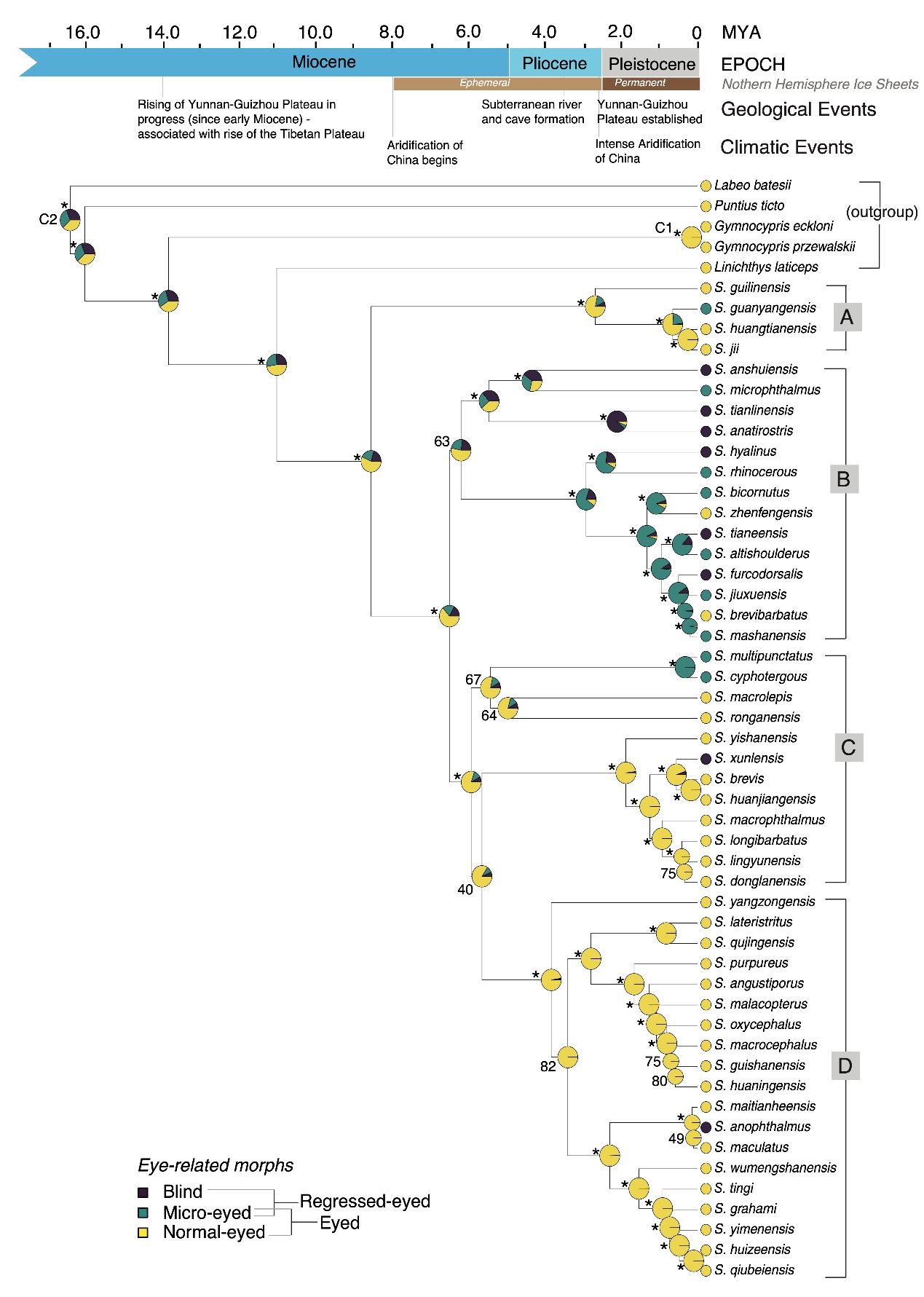
**

**Fig. S1.** Ancestral character state reconstruction using stochastic character mapping for the eye-trait morphology (Blind, Micro and Normal-eyed morphs) on a time calibrated phylogeny. C1 and C2 represent nodes used for calibration of molecular data estimation.


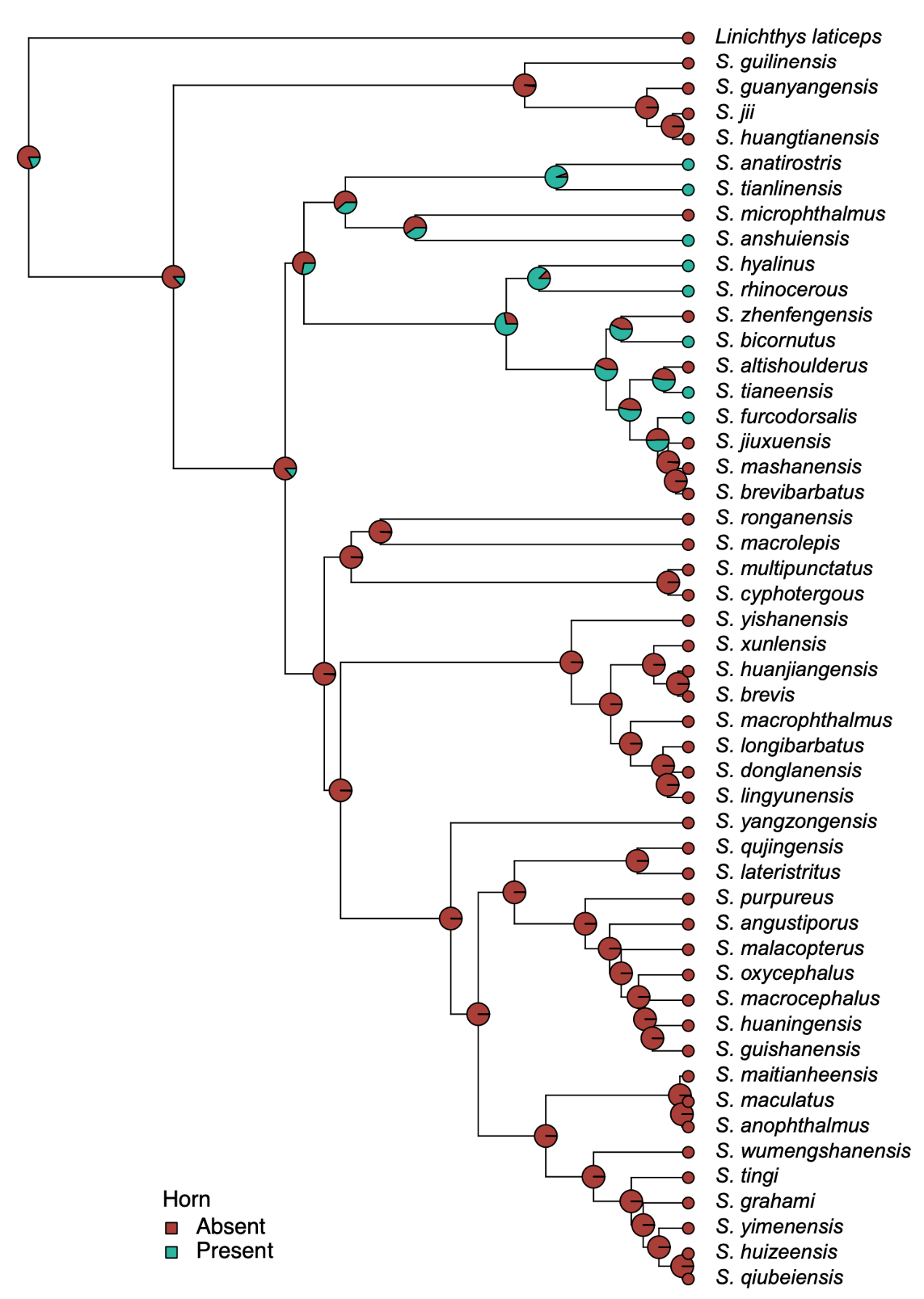


**Fig. S2**. Ancestral character state reconstruction using stochastic character mapping for the horn related trait (presence/absence of horn) on the phylogeny of the genus *Sinocyclocheilus.*


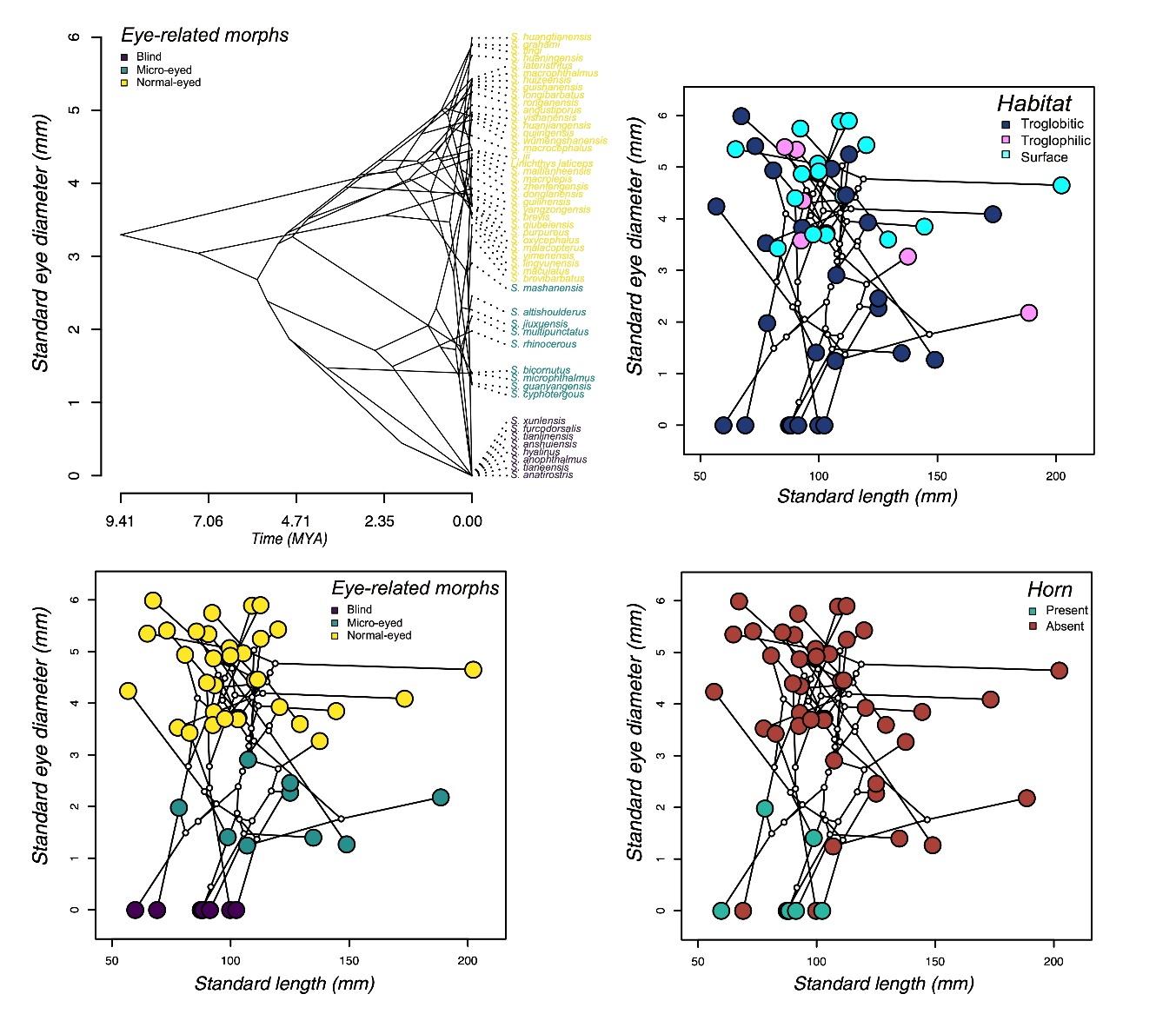


**Fig. S3**. **Temporal patterns of eye diameter evolution and the other three standardized traits (Horn, Habitat, Eye-related morphs) against standard lengths as phylomorphospace-traitgrams in *Sinocyclocheilus*.** These traitgrams suggests that the evolution of different morphs is attained by altering the allometric relationships between body size and eye diameter. (A) Traitgram of standardized eye-diameter shows multiple independent origins of blind forms from early-Miocene to Pleistocene and that Normal-eyed forms were early emerging; (B) Eye related morphs traced on the phylomorphospace indicating clear separation of the three morphs in the morphospace. Blind forms always have a small body size. (C) Habitat associations traced on the phylomorphospace showing species having eye diameter <3mm and small to medium body sizes are obligate cave dwellers whereas species with eye diameter ≥3mm can be Troglobitic, Troglophilic or Surface dwellers regardless of their body size. (D) Horn related morphs traced on the phylomorphospace indicating the presence of a horn in smaller blind fish and smaller fish with reduced eye size. Horned species are all Troglobites.­­


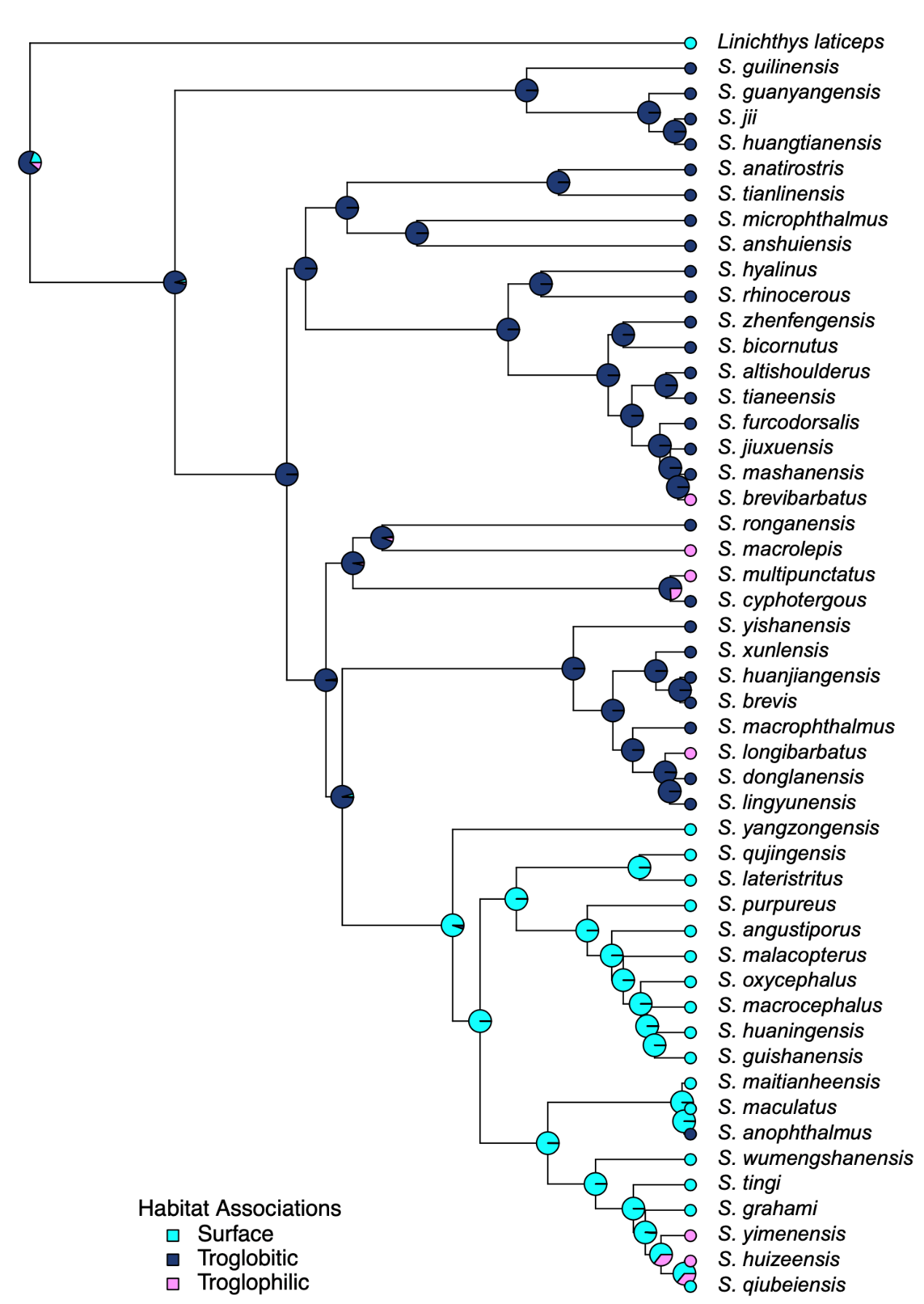


**Fig. S4.** Ancestral character state reconstruction using stochastic character mapping for habitat occupation (Troglobitic, Troglophilic and Surface) on the phylogeny of the genus *Sinocyclocheilus*.

**Table S1**. Calculated Principal Component values (PC1, PC2 and PC3) of all the specimens used in the current analysis

| Species name | PC1val | PC2val | PC3cal |
| --- | --- | --- | --- |
| *Linichthys laticeps* | -0.1069618 | -0.006044939 | -0.032010383 |
| *S. altishoulderus* | 0.01804998 | -0.010081536 | -0.046423134 |
| *S. anatirostris* | 0.01766386 | 0.046175863 | 0.051169448 |
| *S. angustiporus* | -0.04334953 | -0.002675304 | 0.034186136 |
| *S. anophthalmus* | -0.02908059 | 0.031606384 | 0.00917709 |
| *S. anshuiensis* | 0.06639039 | -0.000250595 | -0.004873197 |
| *S. bicornutus* | 0.07667729 | 0.060886565 | 0.012973894 |
| *S. brevibarbatus* | 0.05048176 | -0.011045867 | 0.006805293 |
| *S. brevis* | 0.0142754 | -0.011768784 | -0.001763996 |
| *S. guanyangensis* | 4.38177E-05 | 0.015156895 | -0.025200906 |
| *S. cyphotergous* | 0.1220022 | -0.139796596 | 0.008525167 |
| *S. donglanensis* | -0.004198408 | 0.013428914 | -0.021746042 |
| *S. furcodorsalis* | 0.050741 | -0.000898602 | -0.044412032 |
| *S. grahami* | -0.02231859 | 0.026236004 | 0.022098623 |
| *S. guilinensis* | -0.01963493 | -0.0033727 | -0.023009295 |
| *S. guishanensis* | -0.07886827 | 0.00257981 | -0.059167855 |
| *S. huangtianensis* | -0.05562456 | -0.011114954 | 0.021111628 |
| *S. huaningensis* | -0.04117411 | -0.037450418 | 0.031061221 |
| *S. huanjiangensis* | -0.009958768 | -0.021868616 | 0.007063138 |
| *S. huizeensis* | 0.003779219 | 0.010286192 | 0.031012201 |
| *S. hyalinus* | 0.03974731 | 0.030183657 | 0.039671171 |
| *S. jii* | -0.02155773 | -0.019701056 | -0.005284772 |
| *S. jiuxuensis* | 0.06337799 | 0.004932247 | -0.008080491 |
| *S. lateristritus* | -0.01609448 | -0.006156199 | 0.021918263 |
| *S. lingyunensis* | -0.01666848 | 0.015544267 | 0.002003128 |
| *S. longibarbatus* | -0.0238826 | -0.037134912 | 0.007645544 |
| *S. macrocephalus* | -0.0223646 | -0.03930768 | 0.034945546 |
| *S. macrolepis* | -0.002586613 | 0.0056233 | -0.005281302 |
| *S. macrophthalmus* | -0.01921085 | -0.004423179 | 0.011957836 |
| *S. maculatus* | 0.01164108 | -0.003338214 | -0.043022304 |
| *S. maitianheensis* | -0.04058493 | -0.015399253 | -0.004022456 |
| *S. malacopterus* | -0.03369873 | 0.016341473 | 0.002649988 |
| *S. mashanensis* | 0.03299028 | -0.033529198 | -0.005547116 |
| *S. microphthalmus* | 0.02936266 | 0.061223556 | -0.040563771 |
| *S. multipunctatus* | 0.05126049 | 0.014657848 | -0.010821531 |
| *S. oxycephalus* | -0.04140438 | 0.030989746 | -0.036348165 |
| *S. purpureus* | -0.02288238 | -0.045035385 | -0.018448807 |
| *S. qiubeiensis* | -0.03248934 | -0.003880679 | 0.012191068 |
| *S. qujingensis* | -0.000644406 | 0.033555511 | 0.046291334 |
| *S. rhinocerous* | 0.08985807 | 0.03649387 | -0.020510521 |
| *S. ronganensis* | -0.05067357 | -0.004282673 | -0.02013066 |
| *S. tianeensis* | 0.06234116 | 0.02827463 | -0.030564848 |
| *S. tianlinensis* | 0.01905521 | 0.012948686 | -0.00406653 |
| *S. tingi* | -0.01508119 | -0.019031787 | -0.002178747 |
| *S. wumengshanensis* | 0.000171791 | -0.027155405 | 0.003238896 |
| *S. xunlensis* | -0.008420042 | 0.022462118 | 0.047598226 |
| *S. yangzongensis* | -0.02622031 | -0.003887167 | -0.006000758 |
| *S. yimenensis* | 0.01762049 | 0.008265106 | 0.019258774 |
| *S. yishanensis* | -0.007505367 | -0.029610044 | -0.01952727 |
| *S. zhenfengensis* | -0.01938822 | -0.002301361 | 0.003044235 |

**Table S2**. Species information and GenBank accession numbers of two mtDNA fragments (*NADH4* and *cytb*) of 49 *Sinocyclocheilus* species. The information of 5 outgroup species is also indicated along with calculated standard length (SL), eye diameter (ED), standard eye diameter (sED), discrete trait categories related to eye morphology (Blind, Micro-eyed, Normal eyed), presence or absence of a horn and habitat occupation (Troglobitic, Troglophilic and surface) for all species used in the analysis. Sequences generated and deposited in Genbank by us are indicated with an *.

| **Species name** | **SL** | **ED** | **SED** | **CYTB** | **NADH4** | **Morph** | **Habitat** | **Horn** |
| --- | --- | --- | --- | --- | --- | --- | --- | --- |
| *Linichthys laticeps* | 110.20 | 4.91 | 4.45 | AY854739 | AY854796 | Normal-eyed | Surface | Absent |
| *S. altishoulderus* | 125.10± 16.74 | 3.07±0.35 | 2.46 | AY854724 | AY854781 | Micro-eyed | Troglobitic | Absent |
| *S. anatirostris* | 88.40 | 0.00 | 0 | AY854708 | AY854765 | Blind | Troglobitic | Present |
| *S. angustiporus* | 99.57±19.79 | 5.05±0.77 | 5.07 | AY854702 | AY854759 | Normal-eyed | Surface | Absent |
| *S. anophthalmus* | 99.80 | 0.00 | 0 | AY854715 | AY854772 | Blind | Troglobitic | Absent |
| *S. anshuiensis* | 87.40 | 0.00 | 0 | NC_027169 | NC_027169 | Blind | Troglobitic | Present |
| *S. bicornutus* | 98.80 | 1.39 | 1.41 | AY854730 | AY854787 | Micro-eyed | Troglobitic | Present |
| *S. brevibarbatus* | 137.55±18.17 | 4.50±0.84 | 3.27 | *MT373106 | *MW548423 | Normal-eyed | Troglophilic | Absent |
| *S. brevis* | 92.91±6.39 | 3.55±0.14 | 3.83 | *MT373105 | *MW548424 | Normal-eyed | Troglobitic | Absent |
| *S. guanyangensis* | 148.92±7.39 | 1.90±1.14 | 1.27 | *MT373108 | *MW548426 | Micro-eyed | Troglobitic | Absent |
| *S. cyphotergous* | 106.95±13.08 | 1.34±0.61 | 1.25 | AY854711 | AY854768 | Micro-eyed | Troglobitic | Absent |
| *S. donglanensis* | 173.37±15.94 | 7.09±0.69 | 4.09 | AB196440 | *MW548425 | Normal-eyed | Troglobitic | Absent |
| *S. furcodorsalis* | 91.29±16.36 | 0.00 | 0 | AY854709 | AY854766 | Blind | Troglobitic | Present |
| *S. grahami* | 112.60 | 6.64 | 5.9 | AY854694 | AY854751 | Normal-eyed | Surface | Absent |
| *S. guilinensis* | 120.68±15.04 | 4.74±0.33 | 3.93 | *MT373104 | *MW548427 | Normal-eyed | Troglobitic | Absent |
| *S. guishanensis* | 64.90 | 3.48 | 5.35 | AY854722 | AY854779 | Normal-eyed | Surface | Absent |
| *S. huangtianensis* | 67.32±20.48 | 4.03±0.72 | 5.99 | *MT373109 | *MW548428 | Normal-eyed | Troglobitic | Absent |
| *S. huaningensis* | 92.20 | 5.30 | 5.75 | AY854718 | AY854775 | Normal-eyed | Surface | Absent |
| *S. huanjiangensis* | 80.79±17.68 | 3.99±0.45 | 4.94 | *MT373103 | *MW548429 | Normal-eyed | Troglobitic | Absent |
| *S. huizeensis* | 85.60 | 4.61 | 5.39 | NC_044072 | NC_044072 | Normal-eyed | Troglophilic | Absent |
| *S. hyalinus* | 59.80±28.43 | 0.00 | 0 | AY854721 | AY854778 | Blind | Troglobitic | Present |
| *S. jii* | 111.43±17.21 | 4.97±0.47 | 4.46 | AY854727 | AY854784 | Normal-eyed | Troglobitic | Absent |
| *S. jiuxuensis* | 125.10 | 2.84 | 2.27 | AY854736 | AY854793 | Micro-eyed | Troglobitic | Absent |
| *S. lateristritus* | 120.00 | 6.52 | 5.43 | AY854703 | AY854760 | Normal-eyed | Surface | Absent |
| *S. lingyunensis* | 77.69±9.35 | 2.75±0.60 | 3.53 | AY854691 | AY854748 | Normal-eyed | Troglobitic | Absent |
| *S. longibarbatus* | 90.63±1.82 | 4.84±0.24 | 5.34 | AY854714 | AY854771 | Normal-eyed | Troglophilic | Absent |
| *S. macrocephalus* | 202.30 | 9.42 | 4.65 | AY854683 | AY854740 | Normal-eyed | Surface | Absent |
| *S. macrolepis* | 93.30 | 4.06 | 4.35 | AY854729 | AY854786 | Normal-eyed | Troglophilic | Absent |
| *S. macrophthalmus* | 73.16±11.50 | 3.96±0.71 | 5.41 | AY854735 | AY854792 | Normal-eyed | Troglobitic | Absent |
| *S. maculatus* | 82.70 | 2.84 | 3.43 | EU366193 | EU366183 | Normal-eyed | Surface | Absent |
| *S. maitianheensis* | 90.00 | 3.96 | 4.4 | AY854710 | AY854767 | Normal-eyed | Surface | Absent |
| *S. malacopterus* | 129.20 | 4.65 | 3.6 | AY854697 | AY854754 | Normal-eyed | Surface | Absent |
| *S. mashanensis* | 107.36±5.72 | 3.13±0.34 | 2.91 | *MT373107 | *MW548430 | Micro-eyed | Troglobitic | Absent |
| *S. microphthalmus* | 134.89±10.51 | 1.89±0.38 | 1.4 | AY854687 | AY854744 | Micro-eyed | Troglobitic | Absent |
| *S. multipunctatus* | 188.60 | 4.11 | 2.18 | AY854712 | AY854769 | Micro-eyed | Troglophilic | Absent |
| *S. oxycephalus* | 103.10 | 3.80 | 3.69 | AY854685 | AY854742 | Normal-eyed | Surface | Absent |
| *S. purpureus* | 97.60 | 3.61 | 3.7 | EU366194 | EU366177 | Normal-eyed | Surface | Absent |
| *S. qiubeiensis* | 103.30 | 3.84 | 3.72 | EU366195 | EU366181 | Normal-eyed | Surface | Absent |
| *S. qujingensis* | 99.90 | 4.91 | 4.92 | AY854719 | AY854776 | Normal-eyed | Surface | Absent |
| *S. rhinocerous* | 78.20 | 1.55 | 1.98 | AY854720 | AY854777 | Micro-eyed | Troglobitic | Present |
| *S. ronganensis* | 112.72 | 5.92 | 5.25 | NC_032385 | NC_032385 | Normal-eyed | Troglobitic | Absent |
| *S. tianeensis* | 102.34±12.43 | 0.00 | 0 | AY854717 | AY854774 | Blind | Troglobitic | Present |
| *S. tianlinensis* | 87.96±27.97 | 0.00 | 0 | *MT373102 | *MW548431 | Blind | Troglobitic | Present |
| *S. tingi* | 109.00 | 6.42 | 5.89 | AY854701 | AY854758 | Normal-eyed | Surface | Absent |
| *S. wumengshanensis* | 92.80 | 4.52 | 4.87 | NC_039769 | NC_039769 | Normal-eyed | Surface | Absent |
| *S. xunlensis* | 69.01±21.44 | 0.00 | 0 | EU366187 | EU366184 | Blind | Troglobitic | Absent |
| *S. yangzongensis* | 144.50 | 5.56 | 3.85 | AY854725 | AY854782 | Normal-eyed | Surface | Absent |
| *S. yimenensis* | 92.50 | 3.31 | 3.58 | EU366192 | EU366179 | Normal-eyed | Troglophilic | Absent |
| *S. yishanensis* | 105.34±8.15 | 5.23±0.23 | 4.97 | *MT373101 | *MW548432 | Normal-eyed | Troglobitic | Absent |
| *S. zhenfengensis* | 56.78 | 2.41 | 4.24 | MK610342 | MK610347 | Normal-eyed | Troglobitic | Absent |
| *Labeo batesii* | 81.87 | 4.31 | 5.27 | AB238967 | AB238967 | Normal-eyed | Surface | Absent |
| *Puntius ticto* | 37.74 | 2.77 | 7.33 | NC_008658 | NC_008658 | Normal-eyed | Surface | Absent |
| *Gymnocypris przewalskii* | 232.06 | 9.585 | 4.13 | AB239595 | AB239595 | Normal-eyed | Surface | Absent |
| *Gymnocypris eckloni* | 108.54 | 6.48 | 5.97 | AY463522 | EU366186 | Normal-eyed | Surface | Absent |

**Table S3**. Information of digitized images used in the morphometric geometric analysis. Table indicates the voucher number of the specimen used for the analysis and the reference from which the image was obtained. Images photographed during the current study are also indicated with voucher numbers with the code GXU (GXU: Guangxi University, China).

| Species name | Reference | Voucher # |
| --- | --- | --- |
| *Linichthys laticeps* | Zhang E, Fang F. Linichthys: a new genus of Chinese cyprinid fishes (Teleostei: Cypriniformes). Copeia. 2005;2005:61–7. | *IHB 78X6242* |
| *S. altishoulderus* | Guangxi University (this study). | *GXU001, GXU002, GXU003* |
| *S. anatirostris* | Zhang E, Fang F. Linichthys: a new genus of Chinese cyprinid fishes (Teleostei: Cypriniformes). Copeia. 2005;2005:61–7. | *IHB 84VII225* |
| *S. angustiporus* | Guangxi University (this study). | *GXU007, GXU008, GXU009* |
| *S. anophthalmus* | Zhang E, Fang F. Linichthys: a new genus of Chinese cyprinid fishes (Teleostei: Cypriniformes). Copeia. 2005;2005:61–7. | *KIZ865949* |
| *S. anshuiensis* | Gan X, Wu T-J, Wei M-L, Yang J. A new blind barbine species, Sinocyclocheilus anshuiensis sp. nov.(Cypriniformes: Cyprinidae) from Guangxi, China. 2013. | *12070276* |
| *S. bicornutus* | Zhang E, Fang F. Linichthys: a new genus of Chinese cyprinid fishes (Teleostei: Cypriniformes). Copeia. 2005;2005:61–7. | *IHB 12209043-9o5o241* |
| *S. brevibarbatus* | Guangxi University (this study). | *GXU010, GXU011, GXU012* |
| *S. brevis* | Guangxi University (this study).  Zhang E, Fang F. Linichthys: a new genus of Chinese cyprinid fishes (Teleostei: Cypriniformes). Copeia. 2005;2005:61–7. | *GXU013, GXU014*  *IHB12209033-87087496* |
| *S. cyphotergous* | Zhang E, Fang F. Linichthys: a new genus of Chinese cyprinid fishes (Teleostei: Cypriniformes). Copeia. 2005;2005:61–7.  Huang J, Gluesenkamp A, Fenolio D, Wu Z, Zhao Y. Neotype designation and redescription of Sinocyclocheilus cyphotergous (Dai) 1988, a rare and bizarre cavefish species distributed in China (Cypriniformes: Cyprinidae). Environmental Biology of Fishes. 2017;100:1483–8. | *IHB12209040*  *ASIZB 204678* |
| *S. donglanensis* | Guangxi University (this study). | *GXU015, GXU016, GXU017* |
| *S. furcodorsalis* | Guangxi University (this study). | *GXU018, GXU019, GXU020* |
| *S. grahami* | Zhang E, Fang F. Linichthys: a new genus of Chinese cyprinid fishes (Teleostei: Cypriniformes). Copeia. 2005;2005:61–7. | *ASIZB03496* |
| *S. guanyangensis* | Guangxi University (this study). | *GXU004, GXU005, GXU006* |
| *S. guilinensis* | Guangxi University (this study). | *GXU021, GXU022, GXU023* |
| *S. guishanensis* | Zhang E, Fang F. Linichthys: a new genus of Chinese cyprinid fishes (Teleostei: Cypriniformes). Copeia. 2005;2005:61–7. | *Li980514005* |
| *S. huangtianensis* | Guangxi University (this study). | *GXU024, GXU025, GXU026* |
| *S. huaningensis* | Zhang E, Fang F. Linichthys: a new genus of Chinese cyprinid fishes (Teleostei: Cypriniformes). Copeia. 2005;2005:61–7. | *ASIZB79228* |
| *S. huanjiangensis* | Guangxi University (this study). | *GXU027, GXU028, GXU029* |
| *S. huizeensis* | Cheng C, Pan X, Chen X, Li J, Ma L, Yang J. A new species of the genus Sinocyclocheilus (Teleostei: Cypriniformes), from Jinshajiang Drainage, Yunnan, China. Cave Res. 2015;2. | *KIZ2013001246* |
| *S. hyalinus* | Zhang E, Fang F. Linichthys: a new genus of Chinese cyprinid fishes (Teleostei: Cypriniformes). Copeia. 2005;2005:61–7.  He Y, Chen X-Y, Xiao T-Q, Yang J-X. Three-dimensional morphology of the Sinocyclocheilus hyalinus (Cypriniformes: Cyprinidae) horn based on synchrotron X-ray microtomography. 2013. | *KIZ916001*  *Photograph in life* |
| *S. jii* | Zhang E, Fang F. Linichthys: a new genus of Chinese cyprinid fishes (Teleostei: Cypriniformes). Copeia. 2005;2005:61–7.  Waryani B, Dai R-J, Abbasi AR. Anatomical studies of the olfactory epithelium of two cave fishes Sinocyclocheilus jii and S. furcodorsalis (Cypriniformes: Cyprinidae) from China. 2013. | *ASIZB62726*  *Photograph in life* |
| *S. jiuxuensis* | Zhang E, Fang F. Linichthys: a new genus of Chinese cyprinid fishes (Teleostei: Cypriniformes). Copeia. 2005;2005:61–7. | *ASIZB102260* |
| *S. lateristritus* | Zhang E, Fang F. Linichthys: a new genus of Chinese cyprinid fishes (Teleostei: Cypriniformes). Copeia. 2005;2005:61–7. | *IHB12209036-865027* |
| *S. lingyunensis* | Guangxi University (this study).  Zhang E, Fang F. Linichthys: a new genus of Chinese cyprinid fishes (Teleostei: Cypriniformes). Copeia. 2005;2005:61–7. | *GXU030*  *ASIZB 73038* |
| *S. longibarbatus* | Guangxi University (this study). | *GXU031, GXU032, GXU033* |
| *S. macrocephalus* | Zhang E, Fang F. Linichthys: a new genus of Chinese cyprinid fishes (Teleostei: Cypriniformes). Copeia. 2005;2005:61–7. | *IHB12209012-662001* |
| *S. macrolepis* | Zhang E, Fang F. Linichthys: a new genus of Chinese cyprinid fishes (Teleostei: Cypriniformes). Copeia. 2005;2005:61–7. | *IHB12209035-87IV457* |
| *S. macrophthalmus* | Guangxi University (this study). | *GXU034, GXU035, GXU036* |
| *S. maculatus* | Zhang E, Fang F. Linichthys: a new genus of Chinese cyprinid fishes (Teleostei: Cypriniformes). Copeia. 2005;2005:61–7. | *Li870808001* |
| *S. maitianheensis* | Zhang E, Fang F. Linichthys: a new genus of Chinese cyprinid fishes (Teleostei: Cypriniformes). Copeia. 2005;2005:61–7. | *IHB12209039-874001* |
| *S. malacopterus* | Zhang E, Fang F. Linichthys: a new genus of Chinese cyprinid fishes (Teleostei: Cypriniformes). Copeia. 2005;2005:61–7. | *KIZ775831* |
| *S. mashanensis* | Guangxi University (this study). | *GXU037, GXU038, GXU039* |
| *S. microphthalmus* | Guangxi University (this study). | *GXU040, GXU041, GXU042* |
| *S. multipunctatus* | Zhang E, Fang F. Linichthys: a new genus of Chinese cyprinid fishes (Teleostei: Cypriniformes). Copeia. 2005;2005:61–7. | *ASIZB73000* |
| *S. oxycephalus* | Zhang E, Fang F. Linichthys: a new genus of Chinese cyprinid fishes (Teleostei: Cypriniformes). Copeia. 2005;2005:61–7. | *IHB12209013-652047* |
| *S. purpureus* | Zhang E, Fang F. Linichthys: a new genus of Chinese cyprinid fishes (Teleostei: Cypriniformes). Copeia. 2005;2005:61–7. | *IHB12209015-731004* |
| *S. qiubeiensis* | Zhang E, Fang F. Linichthys: a new genus of Chinese cyprinid fishes (Teleostei: Cypriniformes). Copeia. 2005;2005:61–7. | *Li990527002* |
| *S. qujingensis* | Zhang E, Fang F. Linichthys: a new genus of Chinese cyprinid fishes (Teleostei: Cypriniformes). Copeia. 2005;2005:61–7. | *ASIZB78790* |
| *S. rhinocerous* | Zhang E, Fang F. Linichthys: a new genus of Chinese cyprinid fishes (Teleostei: Cypriniformes). Copeia. 2005;2005:61–7. | *ASIZB93907* |
| *S. ronganensis* | Luo F, Huang J, Liu X, Luo T, Wen Y. Sinocyclocheilus ronganensis Luo, Huang et Wen sp. nov., a new species belonging to Sinocyclocheilus Fang from Guangxi (Cypriniformes: Cyprinidae). Journal of Southern Agriculture. 2016;47:650–5. | *20151114001* |
| *S. tianeensis* | Guangxi University (this study). | *GXU043, GXU044, GXU045* |
| *S. tianlinensis* | Guangxi University (this study). | *GXU052, GXU053, GXU054* |
| *S. tingi* | Zhang E, Fang F. Linichthys: a new genus of Chinese cyprinid fishes (Teleostei: Cypriniformes). Copeia. 2005;2005:61–7. | *ASIZB60227* |
| *S. wumengshanensis* | Zhang E, Fang F. Linichthys: a new genus of Chinese cyprinid fishes (Teleostei: Cypriniformes). Copeia. 2005;2005:61–7. | *KIZ82100006* |
| *S. xunlensis* | Guangxi University (this study). | *GXU046, GXU047, GXU048* |
| *S. yangzongensis* | Zhang E, Fang F. Linichthys: a new genus of Chinese cyprinid fishes (Teleostei: Cypriniformes). Copeia. 2005;2005:61–7. | *KIZ6351069* |
| *S. yimenensis* | Zhang E, Fang F. Linichthys: a new genus of Chinese cyprinid fishes (Teleostei: Cypriniformes). Copeia. 2005;2005:61–7. | *Li030509009* |
| *S. yishanensis* | Guangxi University (this study). | *GXU049, GXU050, GXU051* |
| *S. zhenfengensis* | Liu T, Deng HQ, Ma L, Xiao N, Zhou J. Sinocyclocheilus zhenfengensis, a new cyprinid species (Pisces: Teleostei) from Guizhou Province, Southwest China. Journal of Applied Ichthyology. 2018;34:945–53. | *GZNU20120701001* |
| *Puntius ticto* | Sit G, Jana A, Chanda A. Diversity of Small Indigenous Freshwater Ornamental Fish under Genus Puntius from Purba Medinipur, Paschim Medinipur and Jhargram Districts of West Bengal, India. azb. 2020;8:334–41. | *None* |
| *Labeo batesii* | Ratnasingham S, Hebert PDN. BARCODING: bold: The Barcode of Life Data System (http://www.barcodinglife.org): BARCODING. Molecular Ecology Notes. 2007;7:355–64. (http://v3.boldsystems.org/index.php/Taxbrowser_Taxonpage?taxid=106155) | *OS21321 GAB17-1320* |
| *Gymnocypris przewalskii* | Li S, Chen W, Zhan A, Liang J. Identification and characterization of microRNAs involved in scale biomineralization in the naked carp Gymnocypris przewalskii. Comparative Biochemistry and Physiology Part D: Genomics and Proteomics. 2018;28:196–203. | *None* |
| *Gymnocypris eckloni* | Feng X, Jia Y, Zhu R, Li K, Guan Z, Chen Y. Comparative transcriptome analysis of scaled and scaleless skins in Gymnocypris eckloni provides insights into the molecular mechanism of scale degeneration. BMC Genomics. 2020;21:835. | *None* |
